# TobEA: an atlas of tobacco gene expression from seed to senescence

**DOI:** 10.1186/1471-2164-11-142

**Published:** 2010-02-26

**Authors:** Kieron D Edwards, Aureliano Bombarely, Geraint W Story, Fraser Allen, Lukas A Mueller, Steve A Coates, Louise Jones

**Affiliations:** 1 Advanced Technologies (Cambridge) Ltd, 210 Cambridge Science Park, Milton Road, Cambridge, CB4 0WA, UK; 2 Boyce Thompson Institute for Plant Research, Tower Road, Ithaca, New York 14853-1801, USA; 3 Current address: Graduate School of Life Sciences, University of Cambridge, Cambridge, CB2 1RX, UK

## Abstract

**Background:**

Transcriptomics has resulted in the development of large data sets and tools for the progression of functional genomics and systems biology in many model organisms. Currently there is no commercially available microarray to allow such expression studies in *Nicotiana tabacum *(tobacco).

**Results:**

A custom designed Affymetrix tobacco expression microarray was generated from a set of over 40k unigenes and used to measure gene expression in 19 different tobacco samples to produce the Tobacco Expression Atlas (TobEA). TobEA provides a snap shot of the transcriptional activity for thousands of tobacco genes in different tissues throughout the lifecycle of the plant and enables the identification of the biological processes occurring in these different tissues. 772 of 2513 transcription factors previously identified in tobacco were mapped to the array, with 87% of them being expressed in at least one tissue in the atlas. Putative transcriptional networks were identified based on the co-expression of these transcription factors. Several interactions in a floral identity transcription factor network were consistent with previous results from other plant species. To broaden access and maximise the benefit of TobEA a set of tools were developed to provide researchers with expression information on their genes of interest via the Solanaceae Genomics Network (SGN) web site. The array has also been made available for public use via the Nottingham Arabidopsis Stock Centre microarray service.

**Conclusions:**

The generation of a tobacco expression microarray is an important development for research in this model plant. The data provided by TobEA represents a valuable resource for plant functional genomics and systems biology research and can be used to identify gene targets for both fundamental and applied scientific applications in tobacco.

## Background

*Nicotiana tabacum *(tobacco) is a member of the *Solanaceae *family, which also includes tomato (*Solanum lycopersicum*) and potato (*Solanum tuberosum*), amongst other commercially important crop plants. It was originally studied due to its economic importance but came to the fore of plant biology when it was the first plant to be genetically modified in 1983 [1].

Tobacco is an allotetraploid (2n = 4 × = 48) derived from the interspecific hybridisation of two progenitor genomes; a maternal 'S' genome, originating from an ancestor of *N. sylvestris*, and a paternal 'T' genome, most likely originating from a lineage of *N. tomentosiformis *[2-4]. It has a genome of approximately 5 Gb [5], but despite its commercial and scientific importance relatively little is known about the plant's genome sequence. Recent efforts by the Tobacco Genome Inititative (TGI; http://www.pngg.org/cbnp/index.php?option=com_content&task=view&id=16&Itemid=40) have increased the sequence information available for the transcriptionally active regions of the tobacco genome. Some of this information is in the form of Expressed Sequence Tags (ESTs); short, single pass sequence reads derived from complementary DNA (cDNA) libraries, whilst others are methyl filtered Genome Space Sequence Reads (GSRs).

ESTs provide an insight into transcriptionally active genes in a biological sample under a given set of conditions. EST sequencing is relatively expensive and time consuming. Microarrays, however, provide a faster less costly alternative for measuring gene expression for thousands of genes simultaneously that can be more easily and reproducibly applied across a broad range of conditions or treatments to identify genes showing specific expression patterns or responses. It should be noted that microarray measurements provide an estimate of the steady state level of transcripts. They do not capture all of the post-transcriptional or post-translational regulation of genes and as such do not necessarily reflect the expression of a gene in terms of functional protein produced.

Microarrays have been used in other plant species and organisms such as insects and mammals to capture the variation in gene expression in different tissues throughout development [6-10]. These studies show that organs can be differentiated based on their transcriptional signatures, with sets of genes involved in specific biological processes being expressed only in the relevant tissues. Such data sets provide researchers with information on their gene of interest's expression over a broad range of conditions.

Inference of gene function based on co-expression with other annotated genes is a key component of functional genomics. Community-wide projects, such as AtGenExpress (http://www.weigelworld.org/resources/microarray/AtGenExpress/; [11,12]), and the development of centralised microarray service providers and data repositories, such as the Nottingham Arabidopsis Stock Centre (NASC; http://affymetrix.arabidopsis.info), have resulted in a large amount of publicly available expression data for the model plant *Arabidopsis thaliana*. Development of this resource has enabled comparative analyses to be carried out leading to the identification of strongly co-expressed genes and aiding the elucidation of biochemical pathways for example [13,14]. Associated with the broad availability of such data has been the development of both stand-alone and web-based tools to allow researchers to fully interrogate the expression data sets to further their own research [8,15-19].

Transcription Factors play a key role in regulating gene expression and have been identified by comprehensive analyses in a number of plant species [20-22]. Methyl filtered genomic sequence reads have been used to identify 80-90% of the transcription factors in tobacco [23,24]. Functional genomics is based on a guilt-by-association approach, whereby co-expression of a gene of unknown function with other genes known to be involved in a common biochemical or signalling pathway may suggest that the unknown gene is also involved in this pathway. Co-expression of genes can also be used to infer putative transcriptional networks [25]. The identification of transcription factors that act as regulators of specific biochemical or signalling pathways potentially provide prime targets for manipulation to alter the agronomic or biochemical characteristics of the plant. Such manipulation could prove very useful in projects related to secondary metabolite or biofuel production, or in the case of tobacco leaves, reducing the level of metabolites that give rise to toxicants in cigarette smoke.

This study describes the generation of a custom Affymetrix expression array for tobacco and its use in developing the Tobacco Expression Atlas (TobEA). TobEA is a map of gene expression from multiple tissues sampled throughout the life cycle of the tobacco plant and is intended to be used as a reference data set for plant researchers. The expression data is freely available via the Solanaceae Genomics Network (SGN), a web based genomic resource for plants of the Solanaceae family ([26]; http://solgenomics.net/). Several tools have also been developed to facilitate access to information on researcher's genes of interest.

## Results

### Generation of a microarray for tobacco

In order to enable gene expression studies in tobacco, generation of a microarray based on *Nicotiana tabacum *sequences was undertaken. Currently GenBank contains over 250000 tobacco ESTs, however, prior to the initiation of this project less than 30000 sequences were publicly available for tobacco. To increase the quantity of available sequence data 16 cDNA libraries were generated from multiple tissue types of several tobacco varieties, although there was a focus towards libraries originating from variety K326 and leaf samples. Table 1 summarises the variety and tissue of origin, along with the number of ESTs generated from each of the libraries.

**Table 1:** Tobacco EST libraries

Tissue	Variety	Library name	Number of ESTs
Germinated seeds	K326	KG9	4361
Seedlings	K326	KP1	4692
Roots before topping	K326	KR2	4351
Roots after topping	K326	KR3	5288
Leaf before topping	K326	KL4	4540
Leaf after topping	K326	KL5	3755
Leaf after topping	Burley 21	BL12	1938
Leaf after topping	TN86	TL13	3205
Midrib	K326	KN6	5150
Stem	K326	KT7	4844
Flowers	K326	KF8	4422
Trichomes	Samsun	TT1	3183
Trichomes	Burley 21	TT2	1950
Trichomes	K326	TT3	1161
Early senescent leaf	K326	TT4	1691
Late senescent leaf	K326	TT5	2309
Tobacco leaf	K326, Samsun, Burley 21	TT	2145

Table showing the tissue type and tobacco variety sampled in generating 17 EST libraries. The number of ESTs in each library is shown.

One of two methods was applied to minimise redundancy within each library and maximise the number of unique ESTs generated. Five of the libraries (TT1, TT2, TT3, TT4 and TT5) underwent normalisation prior to sequencing ([27]; see methods). This generated 12439 ESTs, of which 10294 (82.76%) could successfully be sorted into their library of origin based on library specific sequence tags. Despite lacking tags, the remaining 2145 sequences still contained valuable sequence information so were maintained as a generic *N. tabacum *leaf EST library (Table 1). The remaining 11 libraries (KP1, KR2, KR3, KL4, KL5, KN6, KT7, KF8, KG9, BL12 and TL13) underwent virtual subtraction to reduce redundancy [28]. The virtual subtraction libraries were generated as part of the European Sequencing of Tobacco (ESTobacco) project and were included in the tobacco unigene set generated by this effort http://www.ESTobacco.info.

The 58969 ESTs generated by this study, along with another 27219 *N. tabacum *sequences available at the time were assembled into a set of 40642 tobacco unigenes. 57270 of the 86188 sequences assembled into 11724 contigs, with the remaining 28918 singleton sequences completing the unigene set, which ranged in length from 81 bp to 6570 bp (Additional file 1: ATC unigene lengths.ppt and Additional file 2: ATC uingene sequences.zip). Functional annotations for the unigenes were taken from the best BLASTX hit of *Arabidopsis thaliana *proteins (*e*-value <1 × 10^-10^), and/or from the BLASTX hit of non-redundant proteins from Genbank using the program Blast2GO. In total 21060 (51.8%) of the unigenes were annotated by either method (Additional file 3: ATC unigene annotations.txt).

The Affymetrix custom design service was used to produce a tobacco expression array based on the tobacco unigene sequences described above. Of the 40642 sequences submitted, 596 were omitted from the array as suitable probes could not be identified. Standard hybridisation and PolyA controls were included on the array together with four species-specific maintenance genes. A small proportion of the sequences were represented by 2 probe sets, resulting in a total of 43768 probe sets present on the array.

### Temporal and spatial changes in tobacco gene expression

**Figure 1:**
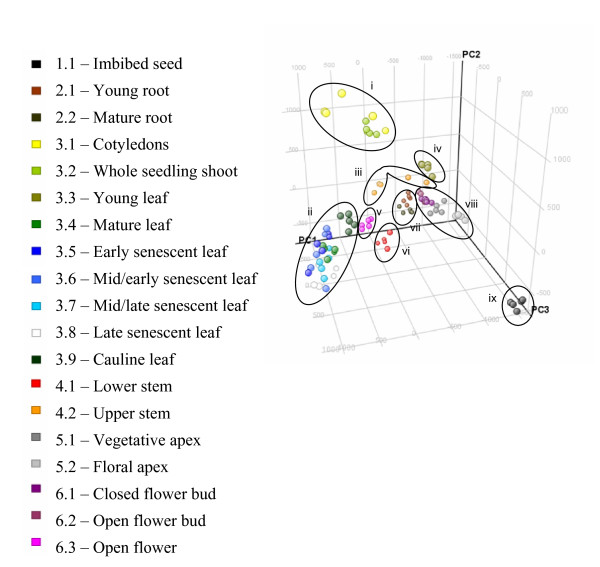
**Tobacco organ expression trends**. Principal Components Analysis (PCA) of the microarray data for the 95 samples in TobEA. PCA is a statistical method that reduces the dimensionality of multidimensional data-sets and enables the variance in gene expression for microarray samples to be represented across a set of vectors. Data points representing individual MAS5 pre-processed tobacco microarray samples are plotted within the first three PC vectors. Samples occupying similar position in PC space share similar gene expression trends. Black oblongs in chart represent clusters of samples occupying similar position in PC space (see inset labels i to ix). Data points are coloured according to sample tissue of origin, with biological replicate samples coloured identically (see inset key for TTI identity).

Temporal and spatial regulation of gene expression plays a critical role in shaping plant growth and development and considerable variation in gene expression at these levels has been demonstrated in several plant species [6,7,9]. Understanding where and when expression occurs provides a valuable insight into gene function and the functioning of the plant as a whole. Using the custom designed Affymetrix expression array, this variation was captured for tobacco genes by measuring gene expression in different tissue types at various stages throughout plant development (see legend in Figure 1 for a full list of the samples). For ease of representation each of the 19 conditions in the Tobacco Expression Atlas (TobEA) was given a Tissue Type Index (TTI) number, with samples from the same tissue/organ grouped together and then ordered temporally based on developmental stage (e.g. sample 1.1 is an imbibed seed and samples 2.1 and 2.2 are roots from a seedling and mature plant respectively).

Data were pre-processed with the MAS5 algorithm to allow the future addition of further tissue types to the TobEA database [29]. Based on the MAS5 flag values, genes were considered expressed if they were called as Present or Marginal in at least four out of the five biological replicates for at least one tissue type. On this basis genes corresponding to 33458 of the 43768 probe sets on the array were expressed. Detection of expression for 76% of the probesets on the array was considered reasonable as TobEA did not comprehensively cover all of the conditions and varietal differences represented in the EST libraries used to generate the sequences that the array was designed against. The detection level is also not much lower than that shown previously in a larger study in Arabidopsis [6]. Of the expressed genes, 30009 showed a significant difference in expression between tissues, based on a one way ANOVA (*p *< 0.05) following the removal of non-changing genes (genes showing expression between + and -1 Log of the median across all conditions). The number of differentially expressed genes identified suggested that expression varied significantly between tobacco tissues and throughout development for the majority of genes in the plant.

Tissues/organs in other plant species can be distinguished based on their transcriptional signature [6,7]. Principal Components Analysis (PCA) was carried out on the TobEA data to determine the relationship between tobacco tissues. Figure 1 shows the individual TobEA microarrays plotted within the first three Principal Components (PCs). For each tissue type, the five biological replicate samples clustered closely together, suggesting that they were a reliable representation of the transcriptional profile for their respective tissue (Figure 1). The 19 different tissue types could be broadly sub-divided into 9 clusters based on their position within PC space (Figure 1). Samples from imbibed seed (Cluster ix) showed distinct separation from the main body of samples along PC3 (Figure 1). The root (Cluster vii) and cotyledon/leaf (Clusters i, ii and iv) samples were also separated along PC3, with the remaining tissue types falling between these groups (Figure 1). This suggested that PC3 was representative of some of the spatial variation in tobacco gene expression.

Some tissue types, such as roots (Cluster vii), could not be distinguished based on the developmental stage, however, other tissue types did show a developmental separation along PCs 1 and 2 (Figure 1). This was apparent for the transition from floral apex to floral buds in Cluster viii (Figure 1). Greater separation in PC space for a developmental series was shown by leaves. Cotyledon and young shoot samples (Cluster i) along with young leaf samples (Cluster iv) showed clear separation from mature and senescing leaf samples (Cluster ii) along PCs 1 and 2 (Figure 1). This showed a clear distinction in transcriptional signature between young and old leaves.

### Gene expression changes during tobacco leaf development

**Figure 2:**
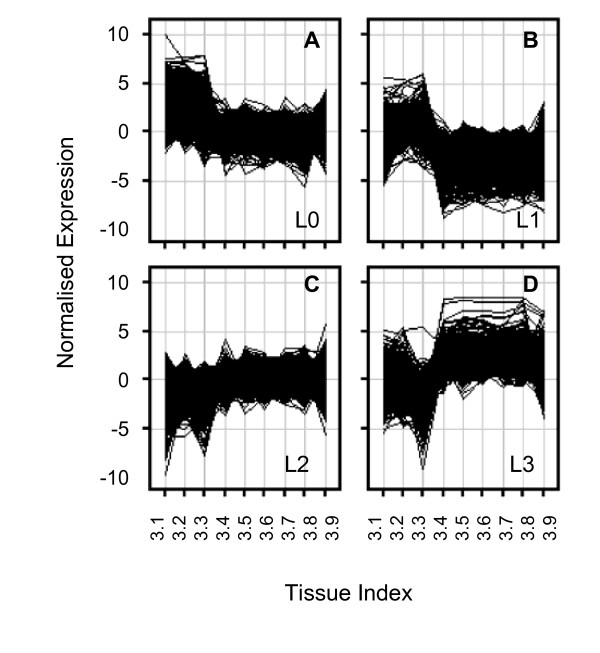
**Changes in gene expression during leaf development**. Genes showing differential expression between young and mature leaf samples were identified (see text for details). These genes were clustered by *K*-means into 4 groups L0 (A), L1 (B), L2 (C) and L3 (D). Expression data are normalised to the median across all 19 tissues in TobEA (y-axis) and plotted against the leaf development samples (TTI 3.1 - 3.9; x-axis).

To investigate the underlying cause of the separation between young and mature/senescing leaf material, genes were identified that showed differential expression across the 'leaf' samples (TTI 3.1-3.9). Genes were called differentially expressed if they showed significant variation (One-way ANOVA *p *< 0.05) and a minimum of five-fold change in expression for at least one pairwise comparison between young (3.1 to 3.3) and mature (3.4 to 3.9) leaf samples. The 8944 genes identified on this basis were clustered by *K*-means into 4 groups as shown in Figure 2. Two basic responses were represented by the 4 clusters; genes showing relatively higher expression in younger samples (Clusters L0 and L1; Figures 2A and 2B) and genes showing relatively higher expression in older samples (Clusters L2 and L3; Figures 2C and 2D). Clusters were tested for over-representation of gene functions, based on the annotations borrowed from the best Arabidopsis BLAST hit.

When genes in clusters L0 and L1 were combined, the Gene Ontology (GO) analysis showed over-representation of multiple terms relating to photosynthesis and cell cycle, or cell growth and expansion (Additional file 4: GO analysis of young versus mature leaves.zip). A combined list of the genes in clusters L2 and L3 showed over-representation of GO terms relating to senescence related responses, including break down and re-mobilisation of various metabolites, hormone responses and stress responses (Additional file 4: GO analysis of young versus mature leaves.zip). This suggested that there was a clear distinction between gene expression in young and old leaf material, with the former showing greater activity in photosynthesis, growth and expansion and the latter undergoing senescence.

Although the four clusters were defined by two basic responses, normalisation of the data across all of the tissues in TobEA allowed further distinction of the clusters. For instance, genes in Clusters L0 and L1 both showed relatively higher expression in younger leaves than older leaves, however, in the context of other tissues in the expression atlas, Cluster L0 genes were relatively highly expressed in young leaf samples, whereas Cluster L1 genes showed relatively low expression in older leaf samples (Figure 2A and 2B). GO analysis of the genes in Cluster L0 principally showed terms associated with Photosynthesis, whereas genes in Cluster L1 showed terms related to cell cycle or cell growth and expansion (Additional file 4: GO analysis of young versus mature leaves.zip). Together, this suggested that the differences between young and old leaf tissues can be described as increased photosynthetic activity in younger samples and reduced cell division and growth along with increased senescence in older leaf tissues. This clearly shows the benefit of considering changes in gene expression in the context of all of the other tissue types in the TobEA database and not just between a limited subset of tissue types.

### Tissue specific tobacco gene expression

**Figure 3:**
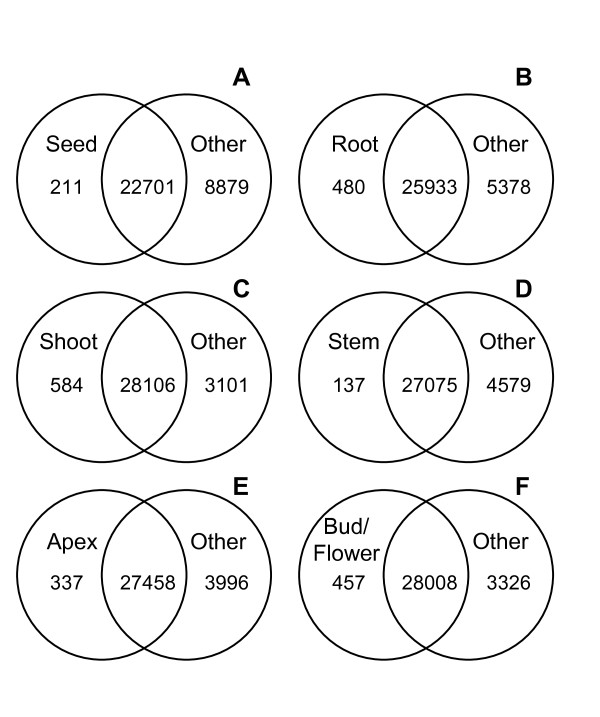
**Tobacco tissue-specific gene expression**. Venn diagram comparing genes identified as expressed in seed (TTI 1; A), root (TTI 2; B), shoot (TTI 3; C), stem (TTI 4; D), apex (TTI 5; E) and bud/flower (TTI 6; F) samples versus those identified as expressed in any of the other 5 tissue types.

The above analyses demonstrated that many tobacco genes show changes in expression between different tissues. To test for genes that are only expressed in specific tissues, all of the expressed genes were identified for each tissue type. For this analysis only genes flagged as Present or Marginal in all 5 biological replicates of at least one developmental stage within a tissue type were considered expressed. Figure 3 shows a Venn diagram for each of the six tissue types represented in the TobEA database, with the genes identified as expressed in each tissue compared to a combined list of all those identified as expressed in the other tissues.

GO analysis was carried out on genes identified as expressed specifically in a single tissue type (Additional file 5: GO analysis of tissue specific genes.zip). The 211 genes identified as specifically expressed in imbibed seed samples (TTI 1.1) showed over-representation of terms associated with seed development and nutrient storage as well as those responsive to water and high light intensity, all processes/responses that are likely to be well represented in an imbibed seed (Additional file 5: GO analysis of tissue specific genes.zip). Similarly, genes specifically expressed in root samples (TTI 2.1-2.2) showed over-representation of terms related to nutrient uptake, transport and toxin or defence responses. Those specifically expressed in leaf samples (TTI 3.1-3.9) showed over-representation of genes involved in photosynthesis (Additional file 5: GO analysis of tissue specific genes.zip). Additionally, genes specifically expressed in stem (TTI 4.1-4.2), shoot apices (TTI 5.1-5.2) and flower buds/flowers (TTI 6.1-6.3) showed over-representation of genes associated with microtubule development, implicated in the regulation of stem growth [30], Chromosome segregation/DNA metabolism and floral organ development respectively (Additional file 5: GO analysis of tissue specific genes.zip).

### Co-expression of tobacco genes

**Figure 4:**
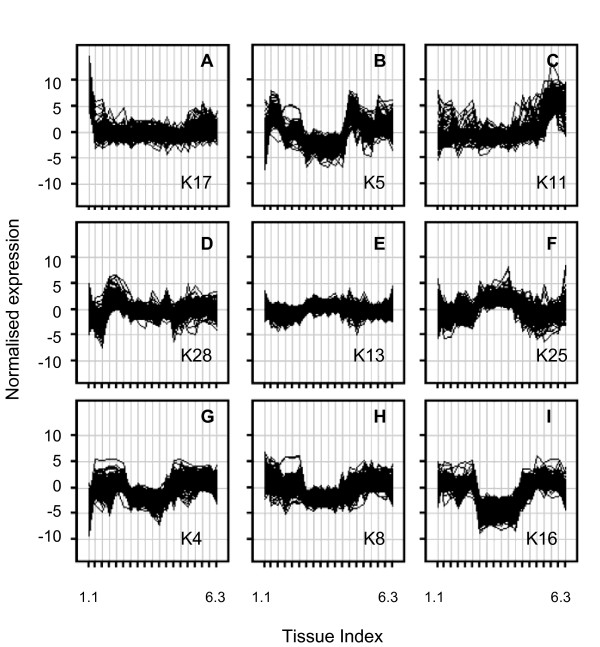
**Tobacco gene co-expression**. 30009 genes identified as differentially expressed across the TobEA samples were *K*-means clustered into 30 groups of co-expressed genes. Panels show normalised gene expression levels (y-axis) plotted across all 19 TobEA tissue types (x-axis) for clusters K17 (A), K5 (B), K11 (C), K28 (D), K13 (E), K25 (F), K4 (G), K8 (H) and K16 (I). The full set of 30 clusters can be seen in Additional file 6: Tobacco gene co-expression.ppt.

The set of 30009 genes showing differential expression across the TobEA samples were analysed for co-expression. Figure 4 shows a subset of 9 clusters from a set of 30 *K*-means clusters (See Additional file 6: Tobacco gene co-expression.ppt and Additional file 7: GO analysis of *K*-means clusters.zip for the complete set of 30 clusters).

GO analysis was carried out on the 30 clusters and many of the identified biological processes could be related to the tissue types they showed expression in. For example genes in Cluster K17 showed relatively high expression in imbibed seed samples (TTI 1.1) and an over-representation of genes involved in seed development and nutrient storage (Figure 4A and Additional file 7: GO analysis of *K*-means clusters.zip). Indeed 50% of the genes in Cluster K17 were among the 211 identified as specifically expressed in imbibed seed. Similarly genes in cluster K11 showed relatively high expression in buds and flowers (TTI 6.1-6.3), and genes in clusters K28 showed relatively high expression in young leaves (TTI 3.1-3.3) and these 2 clusters showed over-representation of genes related to floral development and Photosynthesis respectively (Figures 4C and D and Additional file 7: GO analysis of *K*-means clusters.zip). Another example of expression of genes involved in relevant processes for the respective tissue is offered by Cluster K5. These genes showed high expression in both root and stem samples and over-representation of genes involved in Xylem and Phloem development (Figure 4B and Additional file 7: GO analysis of *K*-means clusters.zip).

Clusters K13/K25 and K4/K8/K16 showed relatively high and relatively low levels of gene expression in mature and senescing leaf samples respectively (Figures 4E-I). The former set of clusters showed over-representation of genes involved in senescence related processes and the latter for genes involved in cell cycle and growth (Additional file 7: GO analysis of *K*-means clusters.zip). Together with the results for cluster K28, this supported the suggestion that transcriptional signature differences between young and old leaf tissues was based on genes involved in photosynthesis, cell division and growth, or senescence.

### Analysis of tobacco transcription factor expression patterns

Transcription factors (TFs) play an important role in the regulation of gene expression. Comprehensive surveys of the TFs present in several other plant species have been undertaken [20-22]. Although complete sequence coverage is not available for the tobacco genome, the GSR sequence data generated by the TGI allowed the identification of 2513 TFs in *N. tabacum *as part of the TOBFAC database [23,24]. A BLASTN search was used to map these TFs to the tobacco unigenes assembled during this study (see methods and Additional file 8: Tobacco transcription factors.txt). The 2513 TFs were mapped to 779 unique unigenes, of which 772 were represented by at least one probe set (850 probe sets in total) on the microarray.

**Figure 5:**
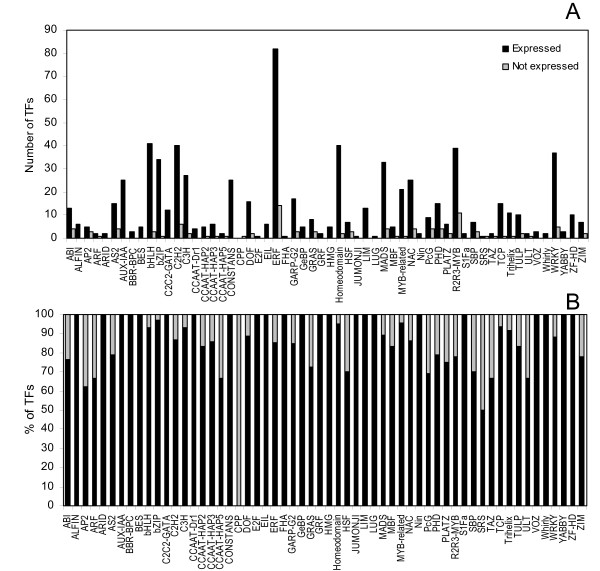
**Tobacco TF expression**. 772 of the 2513 TOBFAC TFs were mapped by BLAST searches to 850 probe sets on the tobacco microarray (See methods). Bar charts show the number (A) and percentage (B) of probe sets representing transcription factors in each TOBFAC family that were identified as expressed in at least one TobEA tissue type. Black bars represent the number/percentage of expressed genes and grey bars the non-expressed genes. TF families are identified along the x-axis.

Of the TOBFAC TFs mapped to probe sets on the array, 87% were identified as expressed in at least one tissue. Figure 5 shows the number and percentage of tobacco microarray probe sets representing TFs for each of the TOBFAC families scored as expressed. For the majority of the TF families over 60% of their members were scored as expressed (Figure 5B). The exceptions to this were the SHI Related Sequence (SRS) and CPP families, where only 50% and 0% were expressed respectively (Figure 5B). However, it should be noted that both these families were represented by two or less probe sets (Figure 5A).

The Ethylene Response Factors (ERF) are one of the largest TF families in Tobacco with over 230 members [24] and are represented by 96 probesets on the tobacco microarray. The ERF family did not show any strong trend in tissue specificity within TobEA, with a large number being expressed in all of the different tissue types (43.8% expressed in seed, 65.6% in root and between 52% and 61% in the other tissue types). The ERFs are divided into 10 subgroups, and a high percentage of group IX tobacco ERFs were previously shown to be induced in response to methyl jasmonate (MeJA) treatment [24]. The Group IX ERFs were represented by 11 probe sets on the tobacco microarray. Compared to the family as a whole, the group IX ERFs showed fewer members expressed in all other tissues (18.2% in seed, 54.5% in leaves, 45.5% in both stem and apices and 36.4% in buds and flowers) aside from roots, where 72.7% of them were scored as expressed. MeJA treatment has been shown to inhibit root growth in Arabidopsis [31] and tobacco [32] and it may be that the induction of these root expressed group IX ERFs by MeJA plays an important role in the inhibition of growth.

### Identification of putative transcriptional networks

**Figure 6:**
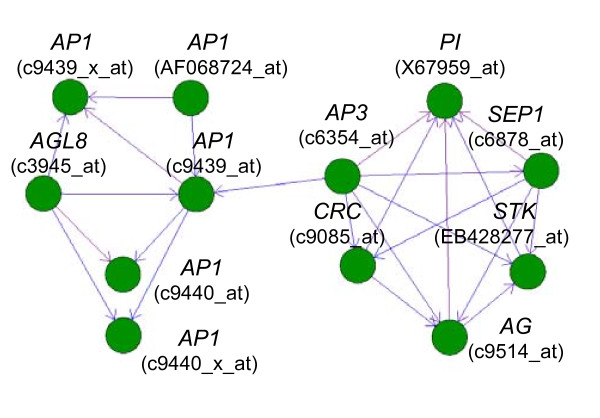
**Tobacco floral identity TF co-expression network**. Expression data for the TOBFAC TFs present on the microarray was used to generate co-expression networks with Biolayout 3D [33]. Figure shows a representative gene network consisting of TFs involved in floral meristem determination and identity. Genes are represented by green nodes. Edges between nodes, indicating an interaction based on co-expression, are represented by arrows. Respective gene and probe names are given for each node.

Although expression could be measured for a majority of TFs across a range of different tissues, this does not mean that their expression levels were similar between tissues. Correlation of expression level across different conditions provides a useful tool for functional genomics. The program Biolayout 3D was used to generate gene networks based on co-expression of TFs across all of the arrays in TobEA [33]. Individual probe sets for TFs are represented by nodes in the network and the edges between nodes represent correlated gene expression. Of the 850 probe sets representing TFs on the array, 297 were placed into 20 networks. Figure 6 shows an example of one of the networks generated including orthologs for 6 MADS-box TFs and one YABBY TF involved in floral meristem identity in other plant species (See Additional file 9: Floral identity gene correlation.doc for table of Pearson correlation values between nodes displayed in network).

It should be noted that an edge between two nodes does not necessarily indicate a causal interaction between these nodes as it could equally be interpreted as co-regulated expression of both nodes by a common regulator. However, several interactions suggested by the tobacco floral identity gene network were consistent with the gene regulatory network proposed for Arabidopsis (Figure 6; [34]). For example putative interactions between *APETALA1 *(*AP1*) and *AP3*, between *AP3 *and *AGAMOUS *(*AG*) and both of these genes with *PISTILLATA *(*PI*) in tobacco are supported by results in Arabidopsis [34]. Some interactions shown in Arabidopsis were not represented in the tobacco TF network, such as a positive interaction between *AP1 *and *PI *and a negative interaction between *AG *and *AP1*. Failure to see the latter interaction is not surprising as the network is based on positive correlation.

The TF network also suggests a positive interaction between *AP1 *and *AGAMOUS LIKE 8 *(*AGL8*), whereas *AGL8 *is reportedly repressed by *AP1 *in Arabidopsis (Figure 6 and [35]). This suggests a different relationship may exist between these genes in tobacco compared to Arabidopsis. Two putative tobacco *AGL8 *orthologs were identified, so it was possible that only one had lost the repression by *AP1*. However, the other *AGL8 *ortholog was placed in a separate small network along with another *AP1 *ortholog (as well as *AGL20*), suggesting that both of the tobacco *AGL8 *orthologs were not repressed by *AP1 *(data not shown).

The tobacco TF network appeared to consist of two distinct sub-clusters, one including *AGL8 *and *AP1 *orthologs and the other including orthologs for *AP3*, *PI*, *AG*, *CRABS CLAW *(*CRC*), *SEPALLATA1 *(*SEP1*) and *SEEDSTICK *(*STK*) (Figure 6). This network structure is consistent with the former two genes being involved earlier on during floral determination, whereas the remaining genes have been implicated later in floral development and floral organ identity [36-42].

### Development of web-based tools for TobEA

The tobacco microarray was designed from a unigene assembly based on 86188 sequences. Since the assembly was carried out there has been a significant increase in the sequence data available for tobacco, with over 250 k ESTs currently available at GenBank. A more recent set of 84602 unigenes was assembled from 239761 *N. tabacum *sequences by the SGN. The SGN also houses NicotianaCyc, a database linking the tobacco unigenes to the MetaCyc biochemical pathways [43]. To benefit from the greater genomic coverage and annotation information, unigenes present on the microarray were mapped to the current SGN unigene assembly (see methods). 41,648 of the probe sets on the microarray could be mapped to 31,929 of the SGN unigenes, indicating approximately 38% coverage of the SGN unigene build by the microarray. Approximately 81% of the mapped SGN unigenes were represented by a unique probe set, and over 99% by 4 probe sets or less.

**Figure 7:**
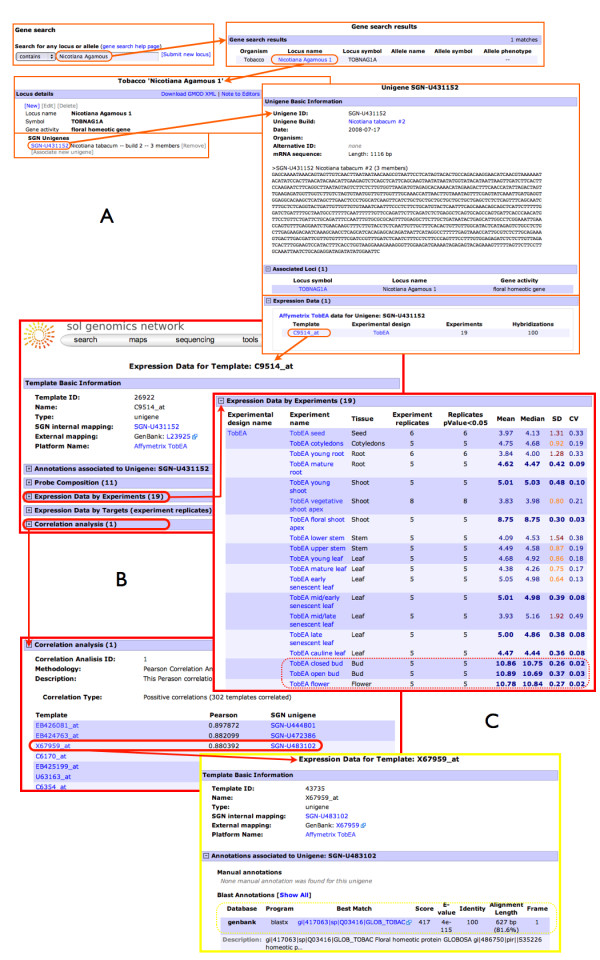
**Data access using SEDM (SGN Expression Data Module)**. Screen shots summarising user access to expression data via the SGN Expression Data Module. Data is available to users from a direct search or via from a search of another SGN element (A). The expression data is shown in the template page with data about the probes, annotation and correlation analysis associated (B). The correlation analysis section can be used to access to other probe-sets with similar expression across the TobEA microarray dataset (C).

Information of the temporal and spatial expression pattern of genes, along with co-expression across a broad range of conditions can be very useful in inferring function for unknown genes. In order to make the information contained within TobEA more accessible to other researchers and enable them to place information on their gene of interest within the context of its expression information, it was decided to integrate the TobEA expression data into the SGN website. Figure 7 shows some sample screen shots of the website, where users can access expression information on their gene of interest. The SGN Expression Data Module (SEDM) allows users to view the expression level of their unigene across the different samples included in TobEA (Figures 7A and 7B). The SEDM also allows users to identify other SGN unigenes that show correlated expression across the TobEA microarray data set (Figure 7C).

## Discussion

Recent efforts, such as the TGI, have massively increased the sequence resources available for *N. tabacum*. However, the 58969 ESTs generated by this study still represent a significant contribution to the total number of ESTs available for tobacco and will prove very useful in applications such as the detection of varietal polymorphisms or gene model predictions. Along with 27219 other available sequences, these ESTs were assembled into a set of 40642 unigenes and used to design a custom Affymetrix expression microarray for tobacco. Affymetrix chips are a proven and widely accepted technology and the design service has been used successfully to generate a large number of custom chips for other research applications http://www.affymetrix.com. The resultant microarray was used to capture the temporal and spatial changes in gene expression throughout the lifecycle of the tobacco plant.

Considerable variation in gene expression was shown across the different tissues within TobEA, with the transcriptional signatures revealing relationships between samples. As observed for Arabidopsis the transcriptional profile of imbibed seed showed the greatest difference to all other samples causing them to be distinctly removed in principal component space [6]. PCA also showed a broad distinction between samples originating from root and shoot tissues. Studies in other plants have shown that the complement of genes expressed in a tissue is related to the biological function that tissue performs [6,7]. Analysis of the GO annotations shows that this is also true in tobacco. As well as spatial changes, temporal changes in gene expression were apparent over developmental series in the same tissues. This was more apparent for some tissues than others. For example PCA showed little separation between young and mature roots. There was, however, a tendency for stronger expression of genes related to cell division, growth and expansion (along with photosynthesis related genes in green samples) in younger tissues and stronger expression of senescence related genes in older tissues. On this basis there was a clear separation between young and old leaf samples, consistent with findings for Arabidopsis [6].

Consistency of the results contained within TobEA with previous results in other plants suggests that it is a high quality expression data set and a good representation of the spatial and temporal changes in gene expression throughout the lifecycle of the tobacco plant. Together with close clustering of independent replicate samples this also indicates that the microarray can be used to reliably and reproducibly measure gene expression in tobacco.

Since the study was initiated larger unigene sets have been assembled for tobacco from the increased amount of sequence data available. These new unigene assemblies likely represent greater coverage of the tobacco genome. Mapping of the microarray probe sets to the current unigene build from the SGN showed that the microarray still represents around 38% coverage of tobacco genes. Mapping of the probe sets to any future unigene assemblies will be maintained to ensure that the annotation information available for the array remains up to date.

As demonstrated by TOBFAC, the tobacco GSR sequence data provides the potential to identify genes that are not represented in the available EST libraries [23,24]. This is most likely the case for rare transcripts or those showing temporal, spatial, or alternative forms of regulation that is not captured in the conditions sampled by the current EST libraries. In future assemblies, the incorporation of sequence data such as GSRs with EST sequences has the potential to provide more comprehensive coverage of the genes present in the tobacco genome than the EST data alone.

The microarray represents a very valuable tool for research in tobacco, allowing comprehensive expression studies to be carried out in the plant. To maximise the benefit of this the array has been made publicly available for use via the Affymetrix service at NASC http://affymetrix.arabidopsis.info. The microarray might also provide a very useful tool to researchers in closely related species, in particular other *Nicotiana *species, via cross-species hybridisations [16,44].

TobEA provides a useful reference data set to any future experiments carried out using the microarray, allowing a more detailed characterisation of the differentially expressed genes identified. To facilitate use of the information contained in TobEA, a set of web-based tools were developed. These tools currently allow researchers to view the expression level of their gene(s) of interest across the different tissues in the atlas and identify other genes that show highly correlated expression via the SGN website. This will enable individual users to carry out functional genomic analysis allowing the annotation of the SGN unigenes to be updated by experts from all different fields of biology via the locus editor feature of the SGN website.

As further experiments are carried out using the tobacco microarray, the data will be uploaded into the SGN expression database SEDM. The SEDM can store results for any Solanaceae species, and is capable of storing data sets from both microarray and direct sequencing studies. Enabling users to upload expression data to the SGN expression database means it has the potential to become a very useful resource for all researchers in the Solenaceae.

The value of TobEA is demonstrated by the study of specific sets of genes such as transcription factors. Due to their regulatory role, TFs represent good targets for gross modifications of biochemical or signalling pathways for various biotechnological applications [45]. Over 30% of the TFs identified by Rushton *et al*., [23,24] in a genome scale survey of tobacco could be mapped to unigenes on the microarray. Simple correlation of TF expression levels across the tissues in TobEA enabled the construction of a gene network involved in floral determination and floral organ identity. Several of the connections observed in this network were consistent with known interactions from other plant species. Extending this type of network analysis to include genes beyond TFs could allow the identification of putative transcriptional networks for specific metabolic or signalling pathways. This analysis could identify regulatory targets to enable the manipulation of these pathways in efforts to increase or decrease the production of various secondary metabolites in tobacco.

## Conclusions

An Affymetrix microarray was developed that allows reliable and reproducible measurement of gene expression in tobacco. This array is available for public use via the NASC Affymetrix service. The array was used to produce the Tobacco Expression Atlas (TobEA), which provides a snap-shot of gene expression from multiple tissues throughout the lifecycle of the tobacco plant. A set of web based tools were developed in order to facilitate public access to this data via the SGN web site http://solgenomics.net/. It is intended that the database will be extended in future with the addition of further expression datasets, not only from tobacco but other plants of the Solanaceae family. The SGN welcomes the donation of such data from the research community. TobEA represents a valuable resource for functional genomic analysis in plants and has potential application in research leading to biotechnological and agronomic improvements to tobacco as well as related species.

## Methods

### Plant materials and growth

Tobacco plants (*Nicotiana tabacum *L. cv. K326; Burley 21; Samsun NN and TN86) were grown on soil (Levington M2), or sand (for root samples) in a greenhouse under a light/dark regime of 16 h light and 8 h darkness. Plants for the EST libraries not required to produce flowers were topped after 12 weeks by removal of the apical meristem. Samples were taken from at least 5 independent replicate plants (30 plants for trichomes, and 1 g for seed) and flash frozen in liquid nitrogen. Leaf senescence was determined empirically by the level of Chlorosis displayed in mature leaves. Chlorophyll levels were measured using a CCM-200 Chlorophyll meter (Opti-sciences Inc., NH, USA), with Chlorophyll Content Index (CCI) readings of between 16 - 25 for early senescent leaves (TTI 3.5), 8 - 16 for mid-early senescent leaves (TTI 3.6), 2 - 8 for mid-late senescent leaves (TTI 3.7) and <2 for late senescent leaves (TTI 3.8). See Additional file 10: TobEA samples.xls for further detail of the growth conditions of individual array samples.

### Preparation of RNA samples

Trichomes were isolated from frozen stems using the method of Aziz *et al*. [46] and RNA extracted using Tri Reagent (Sigma, Gillingham, UK). Total RNA for the remaining cDNA samples was extracted according to the method of Dean *et al*. [47] and mRNA was isolated using DynalBeads (Invitrogen, Paisley, UK). Total RNA was extracted using Trizol for the microarray samples (Invitrogen, Paisley, UK) and purified using RNeasy spin columns (Qiagen, Crawley, UK).

### Sequencing of cDNA libraries

See Additional file 11: Tobacco EST generation.doc for description of the construction and processing of the cDNA libraries. cDNA clones were single pass sequenced from the 5' end using the M13 primer. All sequence information was collated and trimmed for vector sequence. Sequences from the TT1, TT2, TT3, TT4 and TT5 libraries had their 5' sequence tags removed after assignment to their respective libraries. All EST sequences were uploaded to dbEST at NCBI (Genbank accession numbers DV075759, DV157477-DV162736, DV998727-DV998768, DV998776-DV998788, DV998796-DV999999, EB424600-EB452264, EB677167-EB684271, FG633215-FG645637 and FL577778-FL577786).

### Generation of tobacco unigene sequences for microarray design

ESTs were assembled into a unigene set using CAP3 [48] with parameters set as p = 95 and o = 40. Sequences were filtered for polyA tails, low complexity, low quality and short sequences. Sequences were further processed to mask repeat, vector and organelle sequences using EGassembler http://egassembler.hgc.jp. Unigenes were annotated using BLASTX based on the best hit (*e*-value <1 × 10^-10^) against a database of protein sequences from *Arabidopsis thaliana *(The Arabidopsis Information Resource [TAIR] 7.0 release) and also using the program Blast2GO [49] against a database of non-redundant proteins from Genbank.

### Microarray data analysis

Samples were run from 5 independent biological replicates for each tissue type in TobEA. All of the microarray data is available from ArrayExpress (E-MTAB-176). The data set includes an additional 5 microarrays (samples 41, 58, 59, 61 and 79), that were excluded from the statistical analysis of this study to maintain a balanced design, but are present in the SEDM data set. All microarray data were pre-processed using the MAS5 algorithm to facilitate the future addition of further samples to TobEA [29]. Statistical tests were carried out as described in the text using the program GeneSpring ver 10.0 (Agilent, Winnersh, UK) with Benjamini and Hochberg false discovery rate multiple testing correction (MTC; [50]). GO analysis was carried out using a custom Perl script based on GO annotation from the TAIR 8 release (as of April 2009). Only genes with a significant BLASTX hit to Arabidopsis were included in a fishers exact test (with Benjamini and Hochberg MTC [50]) comparing the test list with the whole array as a background list. TF co-expression networks were produced using the tool Biolayout 3D ver 3.0 [33], with a Pearson correlation value of 0.8.

### Mapping of TFs to unigenes on the microarray

TFs identified by Rushton et al., were tested against a custom database of the tobacco unigenes using BLASTN (*e*-value <1 × 10^-10^). Only hits with >75% ID over at least 100 bp were considered. The best hit was selected for each TF based on highest bit score, using the length of the aligned region as a deciding factor where bits scores were equal. Unigene hits for 7 of the TFs could not be distinguished based on the above measure so both unigenes were annotated with the same TF information. Similarly 13 unigenes showed equal hits against two TFs so were annotated with both.

### Mapping of microarray probes to SGN Unigenes

Tobacco Affymetrix probes were mapped to SGN unigenes in a two step process. First, mapping was established between the Genbank accessions or the clone names of the Tobacco Affymetrix probes and the database external references or clone names of the ESTs from the SGN database [26]. Then the SGN unigene members (EST) and SGN unigenes were linked to the Tobacco Affymetrix probes by joining the tables through the common member sequences. The Affymetrix chip contains a number of non-Solanaceae probes, mainly from *Arabidopsis thaliana*, representing positive and negative controls, which obviously were not included in this mapping. Of the 43,768 tobacco probes on the chip, 41,648 could be mapped to 31,929 SGN unigenes.

### SGN Expression Data Module

SEDM was developed to allow users access to TobEA expression data via the SGN website. Data in SEDM are presented for 100 microarray hybridisations with the additional 5 hybridisations provided by extra replicates for some of the 19 conditions as described above. MAS5 pre-processed expression values are presented for SGN unigenes based on mappings from the probe sets as described above. Co-expressed unigenes were identified based on pair-wise Pearson correlation of all probe sets on the microarray using the cor function of R (version 2.7.0) as were the values presented in Additional file 9: Floral identity gene correlation.doc. Only those showing a Pearson correlation co-efficient of <-0.8 or >0.8 are presented.

## Authors' contributions

KDE conceived and initiated the project, carried out bioinformatics analysis and wrote the manuscript. GWS was involved in the initiation of the project and sample processing. FA carried out the sample processing and produced the microarray data. AB and LAM mapped the microarray unigenes to the SGN unigene set and developed the web-based tools. SAC and LJ were involved in the conception and design of the EST sequencing and unigene assembly project and in communication with Affymetrix over the design of the array. All authors were involved in editing of manuscript. All authors have read and approved the final manuscript.

## Supplementary Material

Additional file 1 **ATC unigene lengths**. Histogram showing tobacco unigene lengths (base pairs). Click here for file

Additional file 2 **ATC uingene sequences**. FastA format file containing ATC unigene sequences. Click here for file

Additional file 3 **ATC unigene annotations**. ATC Unigene annotation information file. Genbank IDs of unigene member sequences and information of Arabidopsis and nr best hit information presented for each unigene. Click here for file

Additional file 4 **GO analysis of young versus mature leaves**. Zip file containing lists of probes sets and GO analysis results for genes differentially expressed in young versus mature leaves (cluster L0 to L3). Click here for file

Additional file 5 **GO analysis of tissue specific genes**. Zip file containing lists of probes sets and GO analysis results for genes showing tissue specific expression. Click here for file

Additional file 6 **Tobacco gene co-expression**. Charts showing expression level for genes in *K*-means clusters K0-K29. Click here for file

Additional file 7 **GO analysis of *K*-means clusters**. Zip file containing lists of probes sets and GO analysis results for in each of the 30 *K*-means clusters (K0- K29). Click here for file

Additional file 8 **Tobacco transcription factors**. Table summarising results of mapping TOBFAC TFs to the ATC Unigenes. Click here for file

Additional file 9 **Floral identity gene correlation**. Table showing Pearson correlation values between nodes in the floral gene identity co-expression network. Click here for file

Additional file 10 **TobEA samples**. Table containing information on the biological source of samples contained in TobEA. Click here for file

Additional file 11 **Tobacco EST generation**. Supplementary text with more detailed description of production and processing of EST library samples. Click here for file
