# Supplementary material for: TobEA: an atlas of tobacco gene expression from seed to senescence
Source: BMC Genomics. 2010 Feb 26;11:142. doi: 10.1186/1471-2164-11-142 (PMC2841117; doi:10.1186/1471-2164-11-142)
Supplement: Additional file 1 — ATC unigene lengths. Histogram showing tobacco unigene lengths (base pairs). [file 1471-2164-11-142-S1.PPT]

## Slide 1
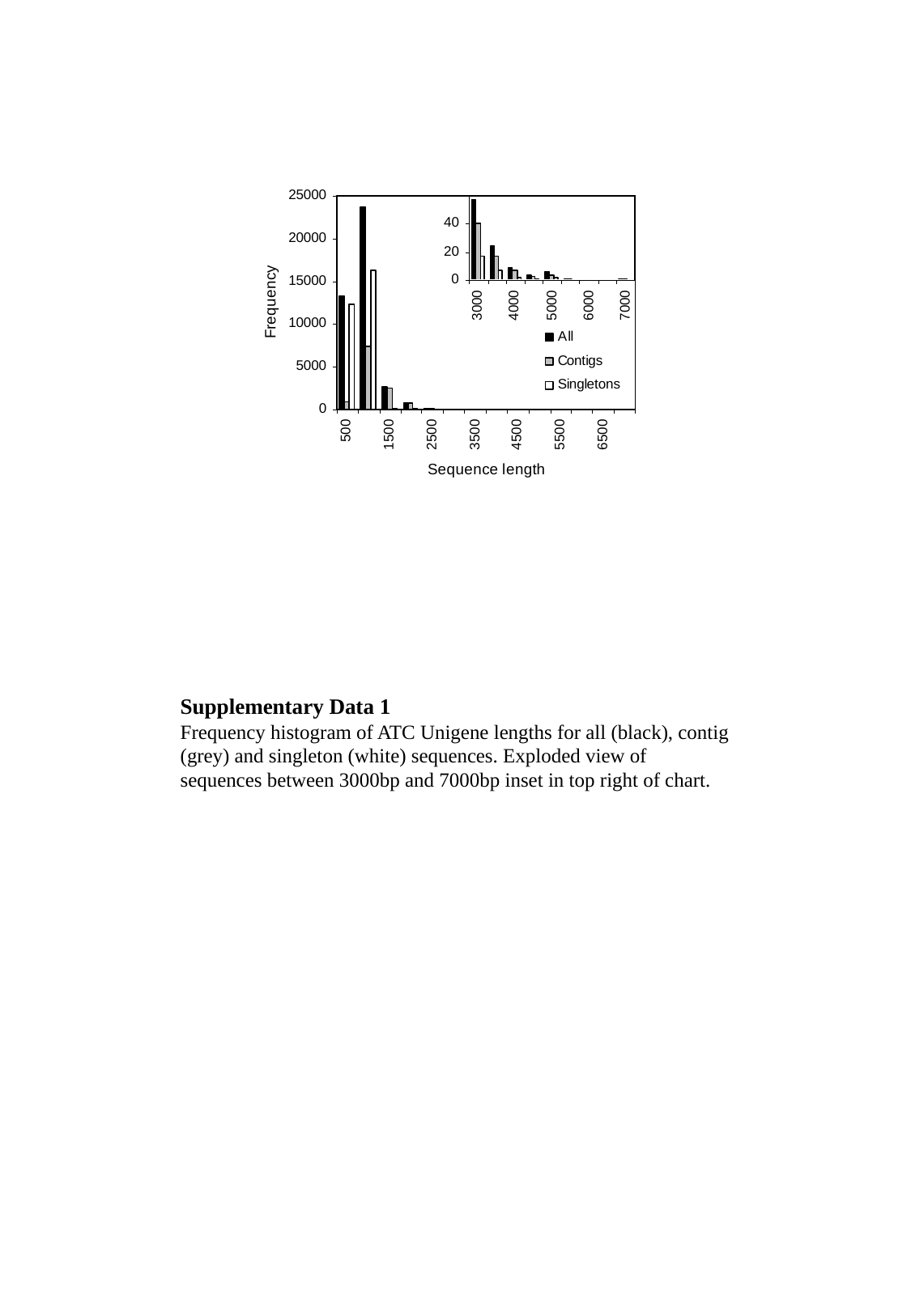

Supplementary Data 1
Frequency histogram of ATC Unigene lengths for all (black), contig (grey) and singleton (white) sequences. Exploded view of sequences between 3000bp and 7000bp inset in top right of chart.
