# Supplementary material for: TobEA: an atlas of tobacco gene expression from seed to senescence
Source: BMC Genomics. 2010 Feb 26;11:142. doi: 10.1186/1471-2164-11-142 (PMC2841117; doi:10.1186/1471-2164-11-142)
Supplement: Additional file 6 — Tobacco gene co-expression. Charts showing expression level for genes in K-means clusters K0-K29. [file 1471-2164-11-142-S6.PPT]

## Slide 1
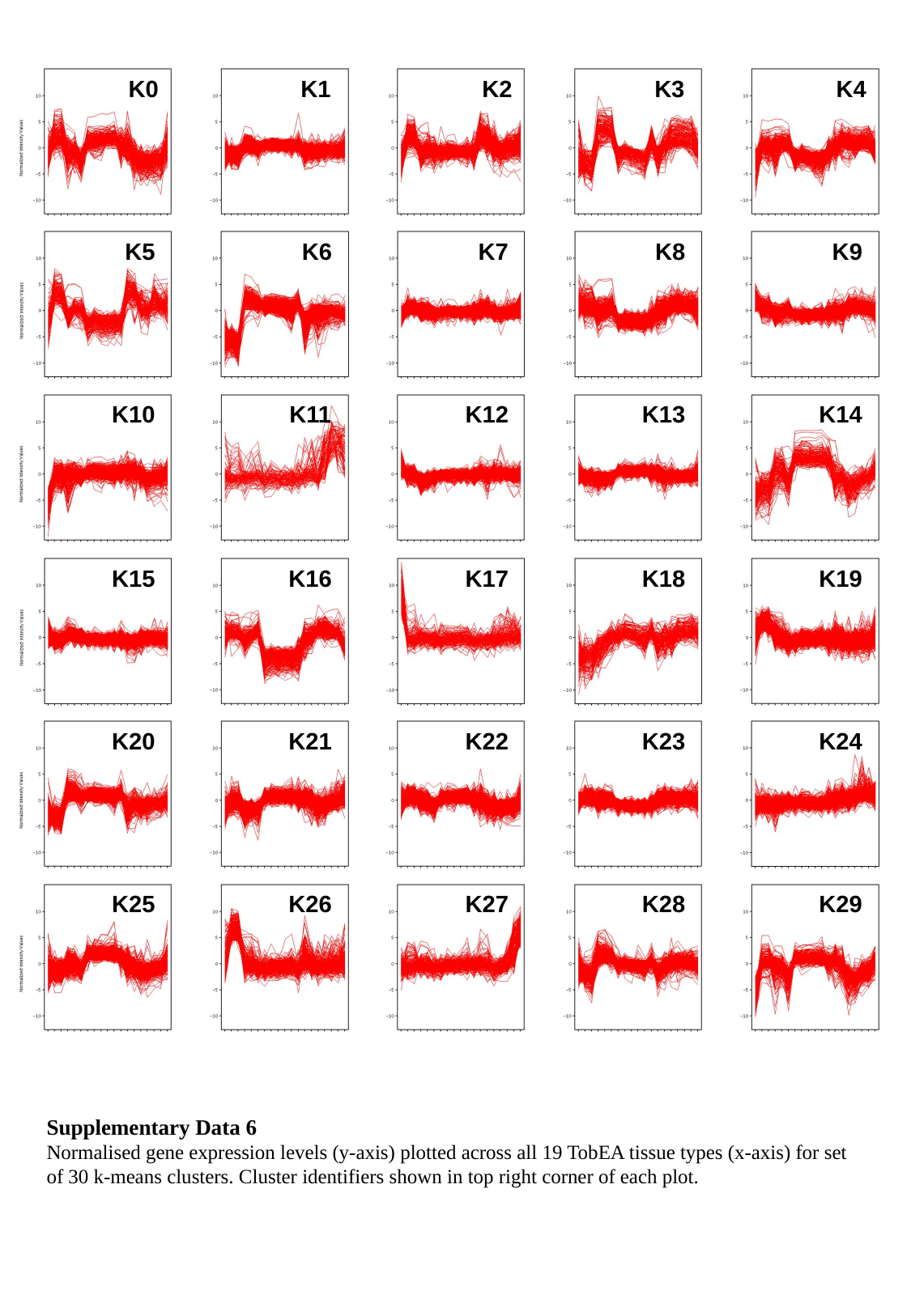

K0
K1
K2
K3
K4
K5
K6
K7
K8
K9
K10
K11
K12
K13
K14
K15
K16
K17
K18
K19
K20
K21
K22
K23
K24
K25
K26
K27
K28
K29
Supplementary Data 6
Normalised gene expression levels (y-axis) plotted across all 19 TobEA tissue types (x-axis) for set of 30 k-means clusters. Cluster identifiers shown in top right corner of each plot.
