# Supplementary material for: TobEA: an atlas of tobacco gene expression from seed to senescence
Source: BMC Genomics. 2010 Feb 26;11:142. doi: 10.1186/1471-2164-11-142 (PMC2841117; doi:10.1186/1471-2164-11-142)
Supplement: Additional file 9 — Floral identity gene correlation. Table showing Pearson correlation values between nodes in the floral gene identity co-expression network. [file 1471-2164-11-142-S9.DOC]

|  | AF068724_at | C3945_at | C9439_at | C9440_at | C9440_x_at | X67959_at | C6354_at | C6878_at | C9085_at | C9514_at | EB428277_at |
| --- | --- | --- | --- | --- | --- | --- | --- | --- | --- | --- | --- |
| C9439_x_at |  |  |  |  |  |  |  |  |  |  |  |
| AF068724_at | 0.86 |  |  |  |  |  |  |  |  |  |  |
| C3945_at | 0.82 | 0.72 |  |  |  |  |  |  |  |  |  |
| C9439_at | 0.87 | 0.82 | 0.82 |  |  |  |  |  |  |  |  |
| C9440_at | 0.80 | 0.75 | 0.83 | 0.80 |  |  |  |  |  |  |  |
| C9440_x_at | 0.79 | 0.72 | 0.89 | 0.82 | 0.79 |  |  |  |  |  |  |
| X67959_at | 0.75 | 0.79 | 0.63 | 0.76 | 0.68 | 0.67 |  |  |  |  |  |
| C6354_at | 0.77 | 0.79 | 0.71 | 0.83 | 0.75 | 0.73 | 0.89 |  |  |  |  |
| C6878_at | 0.72 | 0.75 | 0.57 | 0.71 | 0.58 | 0.58 | 0.88 | 0.85 |  |  |  |
| C9085_at | 0.72 | 0.75 | 0.62 | 0.70 | 0.61 | 0.61 | 0.85 | 0.82 | 0.80 |  |  |
| C9514_at | 0.69 | 0.77 | 0.61 | 0.70 | 0.60 | 0.65 | 0.88 | 0.85 | 0.84 | 0.84 |  |
| EB428277_at | 0.62 | 0.70 | 0.55 | 0.66 | 0.54 | 0.59 | 0.84 | 0.84 | 0.84 | 0.78 | 0.84 |

### Supplementary Data 9

Table showing Pearson correlation values for members of floral gene identity transcription factor co-expression network. Values were calculated using the cor function in R (version 2.7.0).
