# Supplementary material for: TobEA: an atlas of tobacco gene expression from seed to senescence
Source: BMC Genomics. 2010 Feb 26;11:142. doi: 10.1186/1471-2164-11-142 (PMC2841117; doi:10.1186/1471-2164-11-142)
Supplement: Additional file 11 — Tobacco EST generation. Supplementary text with more detailed description of production and processing of EST library samples. [file 1471-2164-11-142-S11.DOC]

# Supplementary text

## Harvesting of plant tissues for cDNA libraries

Material from cv. K326 (unless stated otherwise) was harvested as follows:

Seeds after 2 days imbibition (KG9); two week old seedlings (KP1); roots before topping (KR2); roots one week after topping (KR3); leaf lamina before topping (KL4); leaf lamina one week after topping (KL5); leaf lamina one week after topping from cv. Burley 21 (BL12) amd TN86 (TL13); leaf midrib from all growth stages (KN6); stem from all growth stages (KT7); whole flowers (KF8); trichome-enriched stem samples of cv. Samsun (TT1), Burley21 (TT2) and K326 (TT3); early senescent leaf (TT4); late senescent leaf (TT5).

## Construction and reduction of redundancy in KP1, KR2, KR3, KL4, KL5, KN6, KT7, KF8, KG9, BL12 and TL13 tobacco cDNA libraries

The Smart cDNA library construction kit (Clontech, Mountain View CA, USA) was used for libraries KP1, KR2, KR3, KL4, KL5, KN6, KT7, KF8, KG9, BL12 and TL13. Resulting fragments were ligated into pSOSO2 (a pSPORT1 vector modified to include a multiple cloning site containing an *Sfi*I restriction site). Vectors containing inserts were transformed into *E. coli* strain DH10B and clones picked out into 384 well plates. Approximately 15000 clones were picked in total for each library. The size of insert and quality of the library was investigated by restriction digests of a selected number of clones.

A method of virtual subtraction was applied to obtain a decrease in clone redundancy and an increase in representation of low abundance cDNAs for each library. This was carried out as described previously (Nelson *et al*., 1999) with the adjustments detailed below. In brief, each cDNA library was arrayed onto nylon membranes and grown up until clones were visible but not overlapping. Clones were then lysed and DNA bound in place on the membrane. A random test sequenced subset of radiolabelled cDNAs from the respective library (equivalent to a single 384 well plate of clones from the library) were hybridised to the array. Clones from the array showing no hybridisation were re-arrayed into 384 well plates ready for sequencing. Additionally, there was cross hybridisation of cDNAs from library KN6 onto the KR2 array and reciprocal hybridisation between libraries KT7 and KF8.

## Construction and reduction of redundancy in TT1, TT2, TT3, TT4 and TT5 tobacco cDNA libraries

First strand cDNA synthesis of PolyA^+^ RNA was carried out using an oligo(dT)-*Not* I primer. Adaptor sequences including trimer sequence tags for post sequencing library identification (specific to each cDNA library; see primer sequences below) and an *Eco*RI restriction site at the 5’ end together with a *Not*I restriction site at the 3’ end, were incorporated to the cDNAs during amplification by 10-13 cycles of Long and Accurate (LA) PCR.

Forward primers (trimer sequence tags in bold and underlined):

cDNA library TT1: 5’-GAATTCGTGAGCCAGAGGACGAGACAA**TCG**AA-3’

cDNA library TT2: 5’-GAATTCGTGAGCCAGAGGACGAGACAA**GAG**AA-3’

cDNA library TT3: 5’-GAATTCGTGAGCCAGAGGACGAGACAA**ATG**AA-3’

cDNA library TT4: 5’-GAATTCGTGAGCCAGAGGACGAGACAA**AGC**AA-3’

cDNA library TT5: 5’-GAATTCGTGAGCCAGAGGACGAGACAA**CGG**AA-3’

Reverse primer:

5’-GCGGCCGCTCG(dT25)-3’

N0 cDNA preparations from the five libraries were mixed in a 1:1:1:1:1 ratio. A single cycle of denaturation and reassociation of the mixed cDNAs was carried out to normalize the sample, achieving a Cot-value of approximately 90 in the N1 cDNA sample. Reassociated double stranded (ds) DNA was separated from ssDNA by hydroxyapatite chromatography. ssDNA was amplified with 12 cycles of LA-PCR.

N1 cDNA was directionally cloned into pBluescript II SK (+) vector. Briefly, 5’ *Eco*RI overhangs were generated by limited exonuclease treatment of the N1 cDNA and cleavage by *Not*I at the 3’ end. Fragments were separated by agarose gel electrophoresis and cDNAs greater than 0.5Kb were ligated into the *Eco*RI and *Not*I cleaved vector. Phage T1 resistant TransforMax^TM^ EC100^TM^ electro-competent cells (Epicentre, Madison WI, USA) were transformed with the recombinant vectors by electroporation.
